# Progress on exercise therapy in type 2 diabetes mellitus with cognitive impairment

**DOI:** 10.3389/fspor.2026.1742195

**Published:** 2026-03-24

**Authors:** Yuqiong Xiang, Yulong Zhao, Lin Huang, Junjie Zhou

**Affiliations:** 1 School of Rehabilitation Medicine, Gannan Medical University, Ganzhou, Jiangxi, China; 2 Zhangmu Middle School, Ganzhou, Jiangxi, China; 3 Key Laboratory of Prevention and Treatment of Cardiovascular and Cerebrovascular Diseases of Ministry of Education, Gannan Medical University, Ganzhou, China; 4 Ganzhou Key Laboratory of Rehabilitation Medicine, Ganzhou, Jiangxi, China

**Keywords:** cognitive impairment, diabetes, exercise, therapy, type 2

## Abstract

Patients with type 2 diabetes mellitus (T2DM) face a significantly elevated risk of developing cognitive impairment (CI), which has been recognized as an independent risk factor for dementia. Current glucose-lowering medications are limited by poor central nervous system penetration, delayed intervention, and single-target approaches, highlighting an urgent need for safe and effective complementary strategies. Exercise therapy, leveraging its advantage in “metabolic-neural bidirectional regulation,” demonstrates considerable potential in ameliorating T2DM-related CI. This article systematically reviews basic and clinical research from the past decade, revealing that: ① Aerobic exercise, Tai Chi, and dual-task training can all significantly improve global cognitive scores (MoCA, MMSE), with effect sizes increasing over longer intervention periods; ② Tai Chi yields the most comprehensive benefits in memory, executive function, and balance–fall prevention, with an adherence rate as high as 79.6%; ③ Exercise exerts its effects through multi-target mechanisms, including upregulation of BDNF/IGF-1, suppression of IL-6/TNF-α, restoration of blood-brain barrier integrity, remodeling of the gut microbiota–butyrate–brain axis, and enhancement of mitophagy. Future research should focus on large-sample, multi-center, long-term follow-up studies to establish personalized exercise prescriptions based on genetic–metabolic–microbiota profiles. Integrating digital health technologies will enable remote monitoring and precise implementation, thereby providing an evidence-based foundation for constructing an integrated “metabolic–cognitive” prevention and treatment model.

## 1 Introduction

T2DM is a chronic metabolic disorder characterized by insulin resistance and progressive *β*-cell dysfunction, accompanied by persistent hyperglycemia, dyslipidemia, and multi-organ damage (1). As one of the most critical global public health challenges, the prevalence of T2DM has exhibited an explosive increase over the past four decades. According to the International Diabetes Federation (IDF) 2023 report, approximately 537 million adults worldwide are living with diabetes, more than 90% of whom have T2DM. Without effective interventions, the total number of patients is projected to exceed 783 million by 2045, with the disease burden increasing most rapidly in low- and middle-income countries (136, 137). The pathological essence of T2DM is a systemic collapse of metabolic homeostasis. Driven by the interplay of genetic susceptibility and environmental factors (e.g., obesity, sedentary behavior, high-calorie diet), insulin signal transduction is impaired in skeletal muscle, liver, and adipose tissue, leading to reduced glucose uptake and increased hepatic glucose production. Concurrently, the compensatory insulin secretion by pancreatic *β*-cells progressively fails, ultimately resulting in both fasting and postprandial hyperglycemia. This process intertwines with mechanisms such as chronic low-grade inflammation, mitochondrial dysfunction, and gut microbiota dysbiosis, forming a complex “metabolism-inflammation vicious cycle” (2, 3). Beyond the well-established microvascular (retinopathy, nephropathy, neuropathy) and macrovascular (myocardial infarction, stroke) complications, T2DM is also closely linked to cognitive impairment and has emerged as a significant driver of central nervous system degeneration (4, 5). Robust epidemiological evidence confirms T2DM as an independent risk factor for cognitive decline and dementia: compared to non-diabetic individuals, patients with T2DM exhibit a 60%–90% increased risk of Alzheimer's Disease (AD) and a 2.5-fold higher incidence of vascular dementia. This association remains significant even after adjustment for cardiovascular confounders, suggesting a direct pathophysiological link between hyperglycemia and neuronal injury (6, 7).

The mechanisms underlying T2DM-induced cognitive impairment involve multidimensional interactions. At the metabolic level, chronic hyperglycemia promotes the accumulation of advanced glycation end products (AGEs), triggering neuronal oxidative stress and mitochondrial dysfunction (8). Concurrently, cerebral insulin resistance impairs synaptic plasticity by suppressing the IRS-1/PI3K-Akt signaling pathway and reduces insulin-degrading enzyme (IDE) activity, thereby exacerbating *β*-amyloid (A*β*) deposition (9). In parallel, impairment of the neurovascular unit contributes to the pathology: microvascular endothelial dysfunction disrupts blood-brain barrier integrity, leading to neuroinflammation and white matter hyperintensity (WMH) lesions. Furthermore, reduced cerebral perfusion in hippocampal-prefrontal networks specifically compromises episodic memory and executive function (10). Moreover, T2DM is associated with overlapping neurodegenerative pathologies. The frequent co-occurrence of hyperphosphorylated tau and A*β* pathology in the brains of T2DM patients—a profile highly overlapping with Alzheimer's disease neuropathology—has led to the proposal of “Type 3 Diabetes” (11). Clinical studies indicate that the earliest manifestations of cognitive impairment in T2DM patients are reduced information processing speed and executive dysfunction, which can be detected up to a decade before a formal dementia diagnosis (12). Glycemic variability and severe hypoglycemic episodes accelerate cognitive decline, while current glucose-lowering therapies remain suboptimal for neuroprotection. Although contemporary anti-diabetic agents (e.g., SGLT2 inhibitors, GLP-1 receptor agonists) demonstrate considerable efficacy in controlling metabolic parameters, their protective effects on cognitive function are constrained by three major limitations: limited central penetration, with over 90% of glucose-lowering drugs inadequately crossing the blood-brain barrier to target cerebral insulin signaling pathways; delayed intervention, as pharmacological treatment is often initiated during late-stage neurodegeneration, missing the critical window for pathological reversal; and insufficient multi-target engagement, since single-mechanism glycemic strategies fail to concurrently address core drivers of cognitive impairment such as neuroinflammation and mitochondrial dysfunction (13, 14). Consequently, there is a pressing need to explore safer and more effective therapeutic approaches.

In this context, exercise therapy, leveraging its unique capacity for metabolic-neural bidirectional regulation, emerges as a strategic option to overcome the limitations of current treatments. Basic research confirms that regular physical activity activates the skeletal muscle-brain axis, inducing a surge in circulating neurotrophic factors (e.g., BDNF, IGF-1), enhancing cerebral glucose uptake efficiency, and suppressing A*β* deposition (15, 16). More innovatively, exercise-induced cerebrovascular remodeling can repair the blood-brain barrier damaged by hyperglycemia, while its autonomic nervous system regulatory effects significantly reduce glycemic variability—an independent risk factor for cognition often overlooked by current guidelines. This review aims to systematically elucidate the multidimensional protective mechanisms of exercise therapy in T2DM-related cognitive impairment, establish key components of precision exercise prescriptions based on evidence-based medicine, and prospectively explore its synergistic potential with digital health technologies and pharmacological interventions. The ultimate goal is to provide a theoretical foundation and practical framework for constructing a novel integrated “metabolic-cognitive” prevention and treatment model.

## 2 Pathogenesis of T2DM

### 2.1 Insulin resistance

Insulin resistance, defined as diminished insulin sensitivity in target tissues such as the liver, skeletal muscle, and adipose tissue, represents the initiating event and central mechanism in the pathogenesis of T2DM. During the early stages of T2DM, insulin resistance impairs glucose uptake and utilization in peripheral tissues, triggering compensatory hyperinsulinemia from pancreatic *β*-cells to maintain normoglycemia. As this compensatory mechanism fails, blood glucose levels progressively rise, ultimately leading to the development of overt diabetes (17). Insulin resistance in skeletal muscle represents the earliest pathophysiological alteration in T2DM, accounting for approximately 70%–80% of whole-body glucose disposal. A reduction in insulin-stimulated glucose uptake by skeletal muscle leads to diminished postprandial glucose utilization and contributes to elevated blood glucose levels. This process is closely associated with intracellular lipid accumulation, mitochondrial dysfunction, and activation of inflammatory signaling pathways within skeletal muscle (18). Hepatic insulin resistance is characterized by impaired suppression of hepatic glucose output by insulin, leading to increased hepatic glucose production during fasting and consequent elevated fasting blood glucose. Studies demonstrate that as hepatic insulin resistance worsens, both fasting plasma glucose and 30-minute postprandial glucose levels rise significantly in diabetic patients. Adipose tissue insulin resistance primarily manifests as a reduced ability of insulin to suppress lipolysis, resulting in elevated release of free fatty acids (FFA). The subsequent influx of FFA into non-adipose tissues (e.g., liver and skeletal muscle) further exacerbates systemic insulin resistance, thereby establishing a vicious cycle (19). Impaired insulin signaling constitutes the core molecular basis of insulin resistance. Upon binding to its cell surface receptor, insulin activates receptor autophosphorylation, which subsequently phosphorylates insulin receptor substrates (IRS) and transduces metabolic effects primarily through the PI3K-Akt pathway. Under insulin-resistant conditions, however, serine/threonine phosphorylation of IRS proteins replaces normal tyrosine phosphorylation, thereby disrupting insulin signal transduction (20). Numerous molecules participate in this process, including inflammatory factors (e.g., TNF-α, IL-6), adipocytokines (e.g., decreased adiponectin, leptin resistance), and intracellular stress signals (e.g., JNK, IKK*β*). Recent investigations have highlighted the critical roles of oxidative stress and mitochondrial dysfunction in the development of insulin resistance. Under diabetic conditions, excessive production of reactive oxygen species (ROS) induces oxidative stress, which activates inflammatory pathways and impairs insulin signaling (21). Interestingly, while mild and transient ROS elevation may trigger adaptive antioxidant defenses through “mitohormesis,” chronic and excessive ROS generation exacerbates insulin resistance.

Furthermore, adipose tissue dysfunction and associated inflammation are pivotal factors influencing diabetes. Adipose tissue serves not only as an energy storage organ but also as a significant endocrine organ (22). In obesity-related T2DM, visceral fat accumulation leads to adipocyte hypertrophy and hypoxia, initiating cell death, immune cell infiltration, and chronic low-grade inflammation. Macrophages within adipose tissue polarize towards the pro-inflammatory M1 phenotype, secreting inflammatory cytokines such as TNF-α and IL-6, which disrupt insulin signaling. Notably, interleukin-7 (IL-7) produced by visceral fat is essential for maintaining regulatory T cells (Treg), which themselves help prevent adipose tissue inflammation and thereby suppress the pathogenesis of T2DM (23, 24). In experimental models, interleukin-7 receptor deficiency resulted in a 50% reduction of Treg cells in visceral fat, accompanied by the development of hyperglycemia and insulin resistance (25). This finding reveals a novel interaction mechanism between immune cells and adipose tissue in the pathogenesis of T2DM, offering potential targets for immunotherapeutic interventions.

### 2.2 Islet *β*-cell dysfunction

Defective islet *β*-cell function is a prerequisite for the development of T2DM. Even in the presence of significant insulin resistance, blood glucose levels can be maintained within the normal range as long as *β*-cells compensate by increasing insulin secretion; diabetes becomes clinically manifest only when *β*-cell compensatory mechanisms fail (26). *β*-Cell dysfunction in T2DM progresses through distinct stages: compensation → decompensation → exhaustion. During the compensatory stage, *β*-cells adapt to insulin resistance through hyperplasia, hypertrophy, and enhanced insulin secretion. The decompensation stage is characterized by *β*-cell dedifferentiation, delayed insulin secretory peaks, and aberrant pulse amplitude. In the exhaustion stage, *β*-cell apoptosis and an irreversible reduction in *β*-cell mass occur. Studies have demonstrated that as *β*-cell function declines, significant elevations in fasting blood glucose and postprandial glucose levels at all time points are observed in patients (27). Unlike insulin resistance, which primarily affects fasting and early postprandial glucose, *β*-cell dysfunction impairs glycemic control throughout all postprandial phases, indicating their distinct contributions to dysglycemia in T2DM.

Glucolipotoxicity is a key mechanism driving *β*-cell failure. Chronic hyperglycemia promotes *β*-cell apoptosis via oxidative stress, endoplasmic reticulum stress, and inflammatory responses. Lipotoxicity, resulting from excessive free fatty acids, impairs *β*-cell function through pathways such as ceramide synthesis and mitochondrial dysfunction. Islet amyloid deposition represents a characteristic pathological feature of T2DM, wherein the abnormal aggregation of islet amyloid polypeptide (IAPP) into fibrils exerts direct cytotoxic effects on *β*-cells (28).

### 2.3 Disorders of liver and glucose metabolism

The liver plays a pivotal role in maintaining glucose homeostasis. During the pathogenesis of T2DM, hepatic insulin resistance and increased hepatic glucose output are the primary contributors to fasting hyperglycemia. Under physiological conditions, insulin suppresses glycogenolysis and gluconeogenesis; however, this suppressive effect is markedly attenuated in individuals with T2DM. Hepatic insulin resistance leads to the persistent activation of the FoxO1 transcription factor, which upregulates the expression of key gluconeogenic enzymes such as glucose-6-phosphatase and phosphoenolpyruvate carboxykinase (29). Additionally, diminished hepatic glycogen synthesis capacity further compromises the liver's ability to dispose of glucose.

Hepatic insulin resistance is closely associated with hepatic steatosis, as seen in non-alcoholic fatty liver disease (NAFLD) (30, 31). The deposition of lipids within hepatocytes generates lipotoxic species, including diacylglycerols and ceramides, which activate protein kinase C*ε* (PKC*ε*) and thereby disrupt insulin signaling. Recent studies utilizing liver-specific insulin receptor knockout (LIRKO) mice, which exhibit severe hepatic insulin resistance and glucose intolerance, have provided direct evidence for the autonomous role of hepatic defects in the pathogenesis of T2DM (32).

Patients with T2DM frequently present with dyslipidemia, characterized by increased very-low-density lipoprotein (VLDL) secretion, hypertriglyceridemia, and low high-density lipoprotein cholesterol (33). These alterations are directly linked to hepatic insulin resistance and further exacerbate systemic metabolic dysregulation. The liver acts not only as a target organ for insulin action but also as a central hub for systemic metabolic regulation, communicating with peripheral tissues through the secretion of hepatokines (e.g., FGF21) and metabolites (34, 35).

### 2.4 Immune and inflammatory mechanisms

While T2DM has not been traditionally viewed as an immune-mediated disorder, recent research has progressively uncovered a pivotal role for the immune system in its pathogenesis. Chronic low-grade inflammation serves as a critical link connecting obesity, insulin resistance, and T2DM. Both innate and adaptive immune responses are implicated in the development of T2DM.In adipose tissue, macrophage infiltration and phenotype switching (from anti-inflammatory M2 to pro-inflammatory M1) represent a hallmark feature of obesity-associated insulin resistance (36, 37). M1 macrophages secrete pro-inflammatory cytokines such as TNF-α and IL-6, which activate kinases including JNK and IKK*β*, leading to serine phosphorylation of IRS proteins and subsequent impairment of normal insulin signaling.T lymphocytes also contribute to the regulation of adipose tissue inflammation. An imbalance in the ratio of CD4 + helper T cells (Th1, Th2) and CD8 + cytotoxic T cells promotes a pro-inflammatory microenvironment (38). Notably, regulatory T cells (Tregs) play a crucial role in maintaining immune homeostasis within adipose tissue. Research has revealed that interleukin-7 (IL-7) produced by visceral adipose stromal cells supports Treg survival, thereby mitigating the development of T2DM. Experiments demonstrated that a single administration of interleukin-7 to mice induced a sustained suppression of hyperglycemia, offering a novel perspective for immune-based interventions (39, 40).

Inflammatory cytokines induce insulin resistance through the activation of multiple signaling pathways. The nuclear factor kappa B (NF-*κ*B) pathway serves as a central hub in this process; upon activation by factors such as TNF-α and IL-6, it further amplifies the inflammatory response. The c-Jun N-terminal kinase (JNK) pathway, activated under stress conditions, promotes IRS-1 serine phosphorylation, thereby disrupting insulin signaling (41, 42). Furthermore, inflammasomes—particularly the NLRP3 inflammasome—play a significant role in metabolic inflammation. Activated by metabolic danger signals (e.g., saturated fatty acids, cholesterol crystals), the NLRP3 inflammasome processes and releases IL-1β and IL-18, which directly impair *β*-cell function and insulin sensitivity (43).

Immune-modulatory therapies targeting inflammatory pathways have emerged as a promising direction for T2DM treatment. IL-1β antagonists (e.g., anakinra) have demonstrated modest glucose-lowering efficacy and *β*-cell protective effects in clinical trials (44, 45). In addition, research on interleukin-7 has further advanced the concept of “immunotherapy,” showing potential for developing novel, low-burden therapeutic strategies for patients with T2DM (46). However, the long-term safety of immune interventions, optimal patient selection, and precise dosing strategies require further extensive investigation.

### 2.5 Genetic and epigenetic regulation

T2DM exhibits strong genetic susceptibility, with an estimated heritability ranging from 30% to 70%. Significant progress has been made in understanding the genetic architecture of T2DM in recent years, largely driven by genome-wide association studies (GWAS) and advanced sequencing technologies (2). To date, more than 400 genetic loci associated with T2DM risk have been identified, most of which are implicated in pathways governing pancreatic islet development, insulin secretion, and insulin action.The rs1801282 (Pro12Ala) polymorphism in the PPAR*γ* gene influences adipocyte differentiation, thereby modulating systemic insulin sensitivity (47). In the KCNJ11 gene, which encodes the Kir6.2 subunit of the ATP-sensitive potassium channel, the E23 K polymorphism (rs5219) affects *β*-cell depolarization capacity and insulin secretion, while also being associated with the response to sulfonylurea drugs (48). The vast majority of identified T2DM-associated genetic variants reside in non-coding regions of the genome, where they are postulated to exert their effects by regulating gene expression. While individual variants typically confer only modest effects, their cumulative impact significantly shapes an individual's overall disease susceptibility.

Epigenetic mechanisms—including DNA methylation, histone modifications, and non-coding RNAs—play a pivotal role in gene-environment interactions, helping to explain how environmental factors program metabolic phenotypes. In patients with T2DM, altered DNA methylation patterns have been identified in several key metabolic genes, such as PPARGC1A (a regulator of mitochondrial biogenesis), INS (insulin), and GLUT4 (glucose transporter) (49, 50). Histone modifications also contribute to the phenomenon of metabolic memory, whereby prior periods of hyperglycemia exert long-lasting detrimental effects even after glycemic normalization.Furthermore, non-coding RNAs (e.g., miRNAs, lncRNAs) function as crucial regulators of gene expression in insulin signaling and *β*-cell function. For instance, miR-375 is a key modulator of pancreatic islet development and function (51), while circulating miRNAs show promise as potential biomarkers for T2DM.Beyond these mechanisms, genetic variation influences interindividual differences in responses to glucose-lowering medications. Pharmacogenomics aims to optimize treatment strategies based on genetic profiles. For example, genetic variants in OCT1 (SLC22A1) and MATE1 (SLC47A1) alter the pharmacokinetics of metformin, leading to variations in its plasma concentration. Carriers of the K allele of KCNJ11 rs5219 exhibit a more pronounced reduction in HbA1c following sulfonylurea therapy (e.g., gliclazide), while the PPAR*γ* Pro12Ala polymorphism is associated with an enhanced response to thiazolidinediones (e.g., pioglitazone). Collectively, these findings lay the groundwork for precision medicine in T2DM.

### 2.6 Intestinal Flora and metabolism

The human gut hosts trillions of microorganisms that establish a symbiotic relationship with the host and collectively regulate metabolic homeostasis. Patients with T2DM exhibit gut microbiota dysbiosis, which is characterized by reduced microbial diversity, altered proportions of specific bacterial taxa, and functional disturbances.

The gut microbiota in T2DM exhibits a marked reduction in butyrate-producing bacteria (e.g., Roseburia and Faecalibacterium prausnitzii) and an expansion of opportunistic pathogens (e.g., Bacteroides coprocola and Escherichia coli) (52). This dysbiosis contributes to T2DM pathogenesis through several mechanisms: impaired production of short-chain fatty acids (SCFAs) like butyrate and propionate, which possess anti-inflammatory, insulinotropic, and barrier-protective properties; endotoxemia due to translocated lipopolysaccharide (LPS) from Gram-negative bacteria, promoting systemic inflammation and insulin resistance; and altered bile acid metabolism affecting FXR and TGR5 signaling, thereby disrupting glucose homeostasis and energy expenditure. Through the gut–brain axis, microbial metabolites such as SCFAs stimulate intestinal endocrine cells to release peptides including PYY and GLP-1, which signal via the vagus nerve to the central nervous system and regulate feeding behavior (30, 31, 53). This pathway is impaired in T2DM, leading to disrupted energy balance.Diet rapidly shapes gut microbiota composition and function. A single meal can acutely alter the plasma proteome: ketogenic diets upregulate proteins linked to inflammation, platelet activation, and oxidative stress; high-fat/high-calorie diets elevate adhesion/lipid metabolism proteins and reduce endothelial repair factors; whereas low-fat/low-calorie diets show anti-inflammatory and protective features (54). These insights support precision nutrition as a strategy to modulate microbiota–host crosstalk.

### 2.7 Emerging mechanisms and future directions

#### 2.7.1 Oxidative stress and mitochondrial dysfunction

Oxidative stress and chronic low-grade inflammation are recognized as key drivers of diabetic complications (55). In T2DM, excessive production of reactive oxygen species (ROS) leads to oxidative damage; however, the recently proposed concept of “mitohormesis” has refined the traditional oxidative stress theory—suggesting that mild, transient increases in ROS can trigger adaptive antioxidant defenses, enhance mitochondrial function, and suppress chronic inflammation. This phenomenon has been demonstrated in studies on caloric restriction, exercise, and ketone bodies, underscoring the importance of maintaining a “hormetic window” rather than indiscriminately supplementing antioxidants.

Studies indicate that SGLT2 inhibitor therapy significantly reduces proteinuria and inflammatory markers in patients with diabetic kidney disease, while paradoxically increasing urinary levels of the oxidative stress marker 8-hydroxy-2'-deoxyguanosine and the biological antioxidant potential (BAP), suggesting that its protective effects may involve the induction of an adaptive ROS response (56, 57). Similarly, GLP-1 receptor agonists also modulate oxidative stress markers, implying that multiple metabolic therapies may share common hormetic pathways.

#### 2.7.2 Communication of stress signals between organs

Emerging evidence indicates that stressed adipocytes can transmit mild ROS signals via extracellular vesicles, thereby mediating interorgan mitohormesis. This finding expands our understanding of T2DM as a multiorgan disease and reveals a novel mechanism of interorgan communication (58, 59). Organs such as the liver, adipose tissue, muscle, and gut communicate through the secretion of specific hormones and factors—including hepatokines, myokines, and adipokines—forming an intricate regulatory network. Dysregulation of this interorgan crosstalk underlies the pathogenesis of T2DM and its complications.

#### 2.7.3 Multi-target synergistic intervention strategy

T2DM suggests that multi-target synergistic interventions represent a promising therapeutic strategy. For example, simultaneously targeting the insulin signaling pathway and the inflammatory cytokine IL-1β may improve both insulin resistance and *β*-cell function. Artificial intelligence-assisted target screening is expected to accelerate the establishment of precision therapy systems for diabetes (60). Furthermore, integrating oxidative stress biomarkers—such as urinary 8-hydroxy-2'-deoxyguanosine, reactive oxygen species metabolites, and biological antioxidant potential (BAP)—into clinical monitoring, combined with pharmacological and lifestyle interventions, may facilitate the development of precision metabolic medicine for multi-organ protection in T2DM (61).

## 3 Pathogenesis of T2DM complicated with cognitive dysfunction

**Figure 1:**
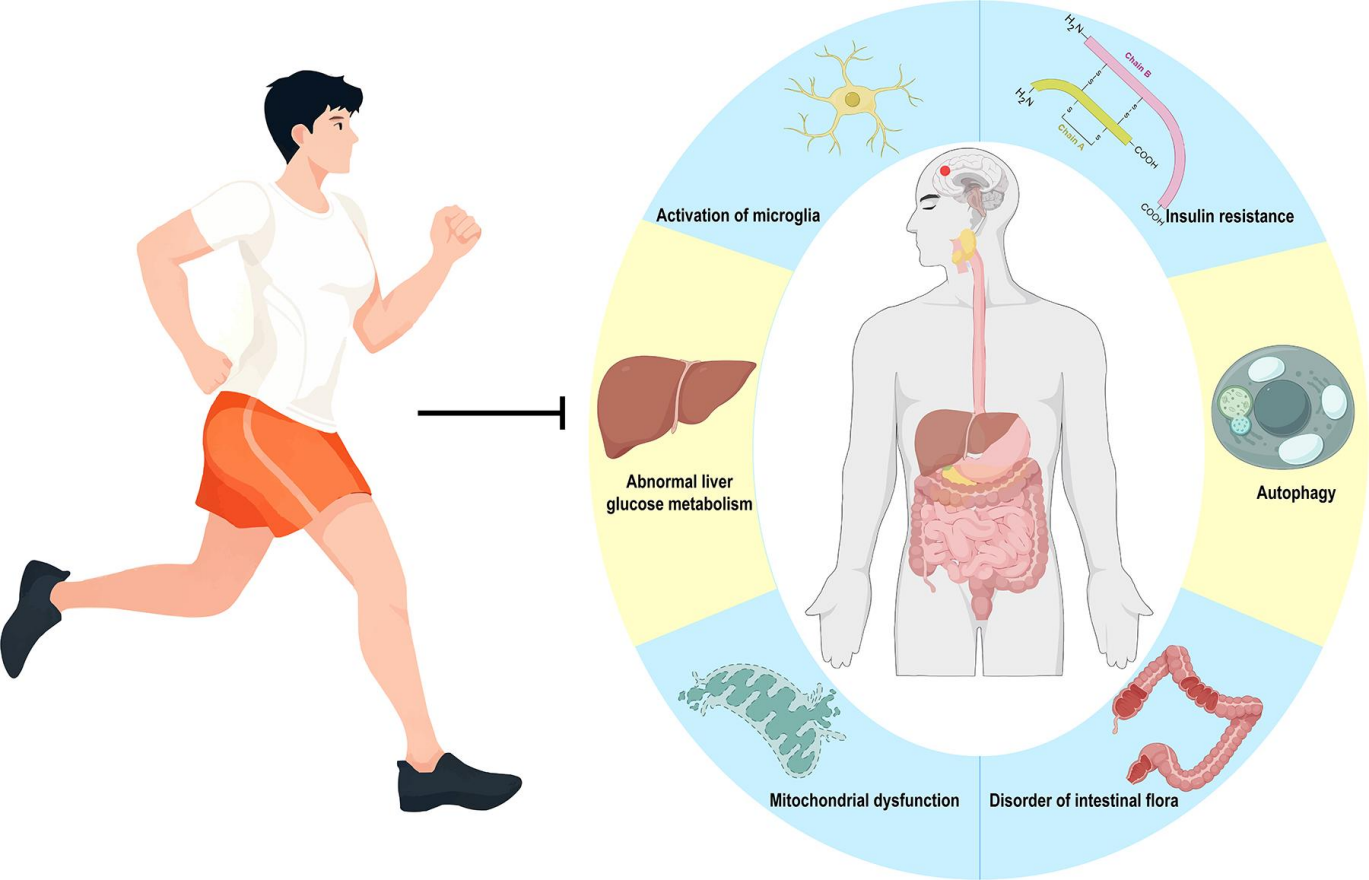
The pathogenesis of T2DM.

A significant proportion of individuals with diabetes ultimately succumb to various complications, among which cognitive dysfunction has garnered increasing attention. The pathogenesis of T2DM-associated cognitive impairment is multifactorial, involving an intricate network of interconnected pathological processes including metabolic disturbances, cerebrovascular impairment, neuroinflammation, and autophagic dysregulation (62) (Figure 1).

### 3.1 Brain insulin resistance and signaling pathway disorders

The brain, as an insulin-sensitive organ, exhibits disrupted insulin signaling that plays a critical role in T2DM-associated cognitive decline (TDACD). Central insulin resistance promotes hyperphosphorylation of tau protein, leading to neurofibrillary tangle formation, while competitively inhibiting insulin-degrading enzyme (IDE), thereby reducing *β*-amyloid (A*β*) clearance and facilitating A*β* deposition (63). The MAPK and PI3-K/Akt signaling pathways, key mediators of insulin-mediated neuroprotection, become dysfunctional in T2DM, exacerbating neuronal injury (64).

Recent neuroimaging studies have identified distinct cerebral glucose metabolic phenotypes in T2DM patients. Using [18 F]FDG-PET, researchers have characterized nine brain regions with consistent metabolic alterations and defined two cerebral metabolic phenotypes: bU[ + ] (predominantly hypermetabolic) and bU[-] (predominantly hypometabolic) (65). The bU[-] phenotype, characterized by reduced glucose uptake in the right superior temporal gyrus, is associated with more severe systemic insulin resistance (HOMA-IR 6.58 vs. 4.03) and elevated levels of the neuroinflammation marker SFRP-1, demonstrating a significant correlation with cognitive impairment risk (66).

### 3.2 Oxidative stress and mitochondrial dysfunction

Oxidative stress serves as a critical link between T2DM and cognitive dysfunction. Under diabetic conditions, systemic overproduction of reactive oxygen species (ROS) shifts the redox balance toward oxidation, ultimately leading to neuronal damage (67). The p66Shc signaling pathway, a key regulator of oxidative stress, plays a pivotal role in TDACD (68). Studies demonstrate that diabetic p66Shc−/− mice exhibit attenuated cerebral oxidative stress, reduced microglial activation, and significantly improved cognitive performance, with this protective effect being at least partially independent of A*β* pathology (69). Mitochondria, functioning as cellular powerhouses and primary ROS sources, rely on a tightly controlled quality control system to maintain neuronal homeostasis. Dysregulation of selective autophagy, particularly mitophagy, results in the accumulation of damaged mitochondria, disrupted energy metabolism, and subsequent synaptic dysfunction and neuronal death in T2DM (70).

### 3.3 Neuroinflammation and microglial cell activation

As Chronic low-grade neuroinflammation represents a core pathological feature of TDACD. Under diabetic conditions, microglia—the resident immune cells of the brain—become aberrantly activated and release pro-inflammatory cytokines such as IL-6 and TNF-α, thereby impairing neuronal function and synaptic plasticity (71). Recent research has demonstrated that shanzhiside methyl ester, an active compound derived from Gardenia jasminoides, alleviates neuroinflammation and modulates glycolytic processes by suppressing the HSP90AA1/HIF1A/STAT1 signaling axis, leading to improved cognitive function in diabetic mice (72). Neuroinflammation is also closely associated with blood-brain barrier (BBB) disruption. Chronic hyperglycemia and elevated inflammatory cytokines in T2DM compromise BBB integrity and increase vascular permeability, facilitating the entry of neurotoxic substances from the circulation into the brain and further exacerbating neuroinflammation and neuronal injury (73, 74).

### 3.4 Selective autophagy and imbalance of protein homeostasis

Emerging evidence highlights the critical role of selective autophagy in maintaining neuronal homeostasis, with its dysfunction being closely implicated in TDACD. Distinct from non-selective autophagy, selective autophagy enables precise degradation of specific organelles or protein aggregates, including mitochondria (mitophagy), endoplasmic reticulum (ER-phagy), and lipid droplets (lipophagy) (75).

Under T2DM conditions, impaired autophagic flux leads to aberrant protein aggregation and organelle dysfunction. For instance, reduced clearance of damaged mitochondria elevates ROS production; insufficient ER-phagy results in sustained activation of the unfolded protein response; while disrupted lipophagy contributes to cerebral lipid accumulation. The dysregulation of these selective autophagy subtypes collectively underpins the pathology of TDACD and offers novel potential therapeutic targets (76, 77).

### 3.5 Cerebrovascular diseases and lymphatic system dysfunction

Microvascular complications induced by T2DM directly impair cerebral blood supply and metabolic waste clearance. Characteristic pathological changes, including basement membrane thickening and pericyte loss, compromise cerebral blood flow regulation and concurrently disrupt glymphatic system function (78). The glymphatic system—a brain-specific waste clearance pathway—relies on the polarized expression of aquaporin-4 (AQP4) water channels in perivascular astrocytic endfeet. In diabetes, mislocalization of AQP4 proteins leads to markedly reduced clearance efficiency, which has been shown to decline by as much as threefold in diabetic rat models (79, 80). This “dual drainage crisis,” combining microangiopathy and glymphatic dysfunction, is recognized as a key mechanism underlying the accumulation of toxic proteins such as A*β* and tau in the brains of T2DM patients, directly contributing to cognitive deficits including spatial memory impairment.

## 4 Treatment strategies for T2DM complicated with cognitive dysfunction

### 4.1 Cognitive protective effects of new hypoglycemic drugs

In recent years, the potential cognitive benefits of novel glucose-lowering agents have attracted considerable attention. Beyond effective glycemic control, medications including glucagon-like peptide-1 receptor agonists (GLP-1RAs), sodium-glucose cotransporter-2 inhibitors (SGLT-2i), and dipeptidyl peptidase-4 inhibitors (DPP4-i) may delay cognitive decline by modulating multiple pathophysiological processes—such as inhibiting inflammation and oxidative stress, reducing apoptosis, decreasing A*β* deposition, and attenuating tau protein phosphorylation.

GLP-1 receptor agonists, which mimic endogenous glucagon-like peptide-1, exert multifaceted neuroprotective effects. Studies indicate that GLP-1RAs enhance mitochondrial function and cellular energy metabolism, thereby lowering reactive oxygen species and alleviating oxidative damage in neurons (81). Furthermore, by activating the PI3 K/Akt/mTOR/GCLc/redox signaling pathway, these agents reduce the expression of apoptosis-related proteins induced by oxidative stress and promote neuronal survival (82). *In vitro* studies also reveal that GLP-1RAs act synergistically with DYRK1A inhibitors to reduce tau hyperphosphorylation via the PI3 K/Akt/GSK3*β* pathway, potentially delaying the onset of Alzheimer's disease (64, 83).Clinical evidence further supports the cognitive benefits of GLP-1RAs. In a comparative study between liraglutide and insulin glargine, liraglutide-treated patients showed significantly higher Montreal Cognitive Assessment (MoCA) scores after one year, along with a lower incidence of hypoglycemia (84). These findings suggest that GLP-1RAs may offer superior advantages over conventional glucose-lowering drugs in preserving cognitive function.

SGLT-2 inhibitors, which lower blood glucose by inhibiting renal tubular glucose reabsorption, have recently been associated with positive effects on cognitive function (85). A prospective study revealed that T2DM patients receiving SGLT-2i in addition to metformin exhibited better performance in cognitive assessments compared to those on metformin alone. Another cognitive evaluation in elderly diabetic patients demonstrated that SGLT-2i significantly improved cognitive impairment, with notably higher Montreal Cognitive Assessment (MoCA) scores in the treatment group than in the control group (86).Preclinical studies have elucidated neuroprotective mechanisms of SGLT-2i, encompassing anti-inflammatory, antioxidant, and mitochondrial function-enhancing effects. For instance, canagliflozin has demonstrated significant antioxidant and anti-inflammatory properties under hyperglycemic conditions (87). It markedly reduced reactive oxygen species and nitric oxide levels in BV-2 microglial cells, while suppressing key inflammatory signaling pathways such as NF-*κ*B, JNK, and p38 MAPK (88).

DPP-4 inhibitors, which exert glucose-lowering effects by preventing the degradation of endogenous GLP-1, have also demonstrated potential benefits in preserving cognitive function (89). In a comparative study between alogliptin and repaglinide, patients treated with alogliptin exhibited significantly higher MoCA scores after 24 weeks, accompanied by a markedly lower incidence of hypoglycemia (90). These findings suggest that DPP-4 inhibitors may offer superior cognitive benefits compared to traditional insulin secretagogues in T2DM patients with mild cognitive impairment.

### 4.2 Application of traditional medicines and Chinese herbal compound prescriptions

In addition to novel glucose-lowering agents, conventional antidiabetic drugs and traditional Chinese medicine (TCM) compounds also hold therapeutic value for T2DM patients with cognitive impairment. Metformin, as a first-line T2DM medication, has been associated with an 8%–10% reduction in dementia incidence in some studies; however, other evidence suggests its cognitive protective effects may be limited, particularly among elderly patients (91).

TCM formulations have also demonstrated unique benefits in treating T2DM with mild cognitive impairment (MCI). In substantial animal studies, numerous traditional Chinese medicine compounds and their constituents have demonstrated promising efficacy against T2DM complicated with cognitive dysfunction (92, 93). Clinical trials have further indicated the therapeutic potential of traditional Chinese medicine in human subjects, though such studies remain limited (94). A clinical study investigating Danxin Jiangtang Tongmai Capsule combined with metformin showed that the combination therapy group achieved significantly higher MoCA and MMSE scores than the metformin-only control group. Additionally, patients in the combination group exhibited greater improvements in glycemic and lipid profiles, as well as in cognition-related biomarkers such as homocysteine and brain-derived neurotrophic factor (95, 96). These findings indicate that integrated traditional Chinese and Western medicine may represent a promising therapeutic direction for T2DM complicated by cognitive dysfunction.

## 5 The role of exercise therapy in T2DM complicated with cognitive dysfunction

### 5.1 Aerobic exercise

Although pharmacological interventions play a significant role in managing T2DM complicated with cognitive dysfunction, the associated side effects often pose substantial challenges for patients. In recent years, numerous clinical trials and meta-analyses have demonstrated the advantages of exercise therapy in ameliorating cognitive impairment in T2DM. As the most extensively studied form of physical activity, aerobic exercise has shown definitive therapeutic effects on T2DM-related cognitive dysfunction. Clinical studies indicate that regular aerobic training significantly improves overall cognitive function in T2DM patients, particularly in the domains of memory, processing speed, and executive function. A randomized controlled trial involving T2DM patients revealed that aerobic exercise intervention led to notable enhancements in Montreal Cognitive Assessment (MoCA) scores and hippocampal volume. These structural improvements were positively correlated with cognitive functional gains, suggesting that aerobic exercise not only ameliorates clinical behavioral performance but may also delay the process of cerebral atrophy (97).

The molecular mechanisms through which aerobic exercise improves cognitive function in T2DM involve multiple pathways. Studies have demonstrated that aerobic exercise significantly upregulates the expression of brain-derived neurotrophic factor (BDNF), which constitutes a central mechanism for its neuroprotective effects. A study involving elderly T2DM patients found that after 24 weeks of aerobic exercise intervention, patients exhibited a marked increase in serum BDNF levels alongside a reduction in methylglyoxal (MG) levels. These changes were significantly correlated with improvements in both immediate and delayed memory (98). In animal studies, T2DM model rats subjected to 8 weeks of aerobic treadmill training showed significantly elevated protein expression of BDNF, nuclear factor erythroid 2-related factor 2 (Nrf2), and heme oxygenase-1 (HO-1) in the hippocampal region. This was accompanied by a decrease in the oxidative stress marker malondialdehyde (MDA) and enhanced activity of superoxide dismutase (SOD) (99). These findings indicate that aerobic exercise likely protects hippocampal neurons by mitigating oxidative stress and enhancing neurotrophic support.

From a metabolic perspective, the improvement in glucose metabolism induced by aerobic exercise in T2DM patients also indirectly supports cognitive health. Research has shown that six months of aerobic exercise significantly reduces fasting blood glucose, glycated hemoglobin (HbA1c), and the insulin resistance index in T2DM patients (100). Enhanced insulin sensitivity may alleviate cerebral insulin resistance, a key pathological mechanism underlying T2DM-associated cognitive impairment. Furthermore, aerobic exercise reduces visceral fat accumulation and decreases the release of pro-inflammatory factors derived from adipose tissue, thereby attenuating systemic low-grade inflammation and its impact on blood-brain barrier permeability.

### 5.2 Resistance movement

As an important form of physical exercise, resistance training demonstrates unique advantages in ameliorating cognitive dysfunction associated with T2DM. Compared to aerobic exercise, resistance exercise exerts its cognitive protective effects through distinct neurobiological mechanisms. A randomized controlled trial involving older adults at risk for T2DM showed that resistance training led to significant improvements in participants' executive function, working memory, and overall cognitive performance, with these improvements correlating with the preservation of hippocampal volume (101). Particularly noteworthy is the pronounced effect of resistance exercise on episodic memory, potentially stemming from its structural impact on the limbic system, especially the hippocampus.

The neuroprotective mechanisms of resistance exercise primarily involve the modulation of neurotrophic factor systems. Studies indicate that resistance exercise significantly increases insulin-like growth factor-1 (IGF-1) levels in T2DM patients. This growth factor not only regulates glucose metabolism but also crosses the blood-brain barrier to promote neurogenesis and synaptogenesis. In T2DM rat models, ladder climbing training (a form of resistance exercise) significantly increased the protein expression of BDNF, HSP70 (102), Nrf2, and HO-1 in the hippocampal region, while reducing oxidative stress levels, demonstrating efficacy comparable to aerobic exercise (103–105). This indicates that resistance exercise similarly possesses potent antioxidant and neurotrophic effects, contributing to the mitigation of T2DM-related hippocampal damage.

Furthermore, resistance exercise provides specific protection for white matter integrity in the brain. Diffusion tensor imaging studies reveal that T2DM patients often exhibit impaired white matter microstructural integrity, which is closely associated with cognitive decline. Resistance exercise can significantly improve white matter integrity in T2DM patients, particularly in the corpus callosum and prefrontal-limbic circuits. These structural improvements are significantly correlated with enhanced executive function and memory (106). This protective effect may originate from the positive influence of resistance exercise on vascular function and cerebral hemodynamics, thereby increasing perfusion and nutrient supply to white matter regions.

### 5.3 Tai Chi

Tai Chi, a traditional Chinese mind-body exercise, demonstrates unique value in improving cognitive function in patients with T2DM. Unlike physical training, Tai Chi integrates multiple elements including physical movement, breath regulation, and cognitive engagement, potentially addressing T2DM-related cognitive impairment through multifaceted mechanisms. A study investigating the impact of telemedicine-based Tai Chi combined with wearable device monitoring on cognitive function in elderly T2DM patients revealed significant findings. Through a three-arm randomized controlled trial, participants were divided into usual care, fitness walking, and Tai Chi groups for a 12-week intervention. The results showed that the Tai Chi group demonstrated significantly greater improvement in the Montreal Cognitive Assessment (MoCA) compared to both the fitness walking and usual care groups. Additionally, the Tai Chi group exhibited superior performance in secondary cognitive indicators including Memory Quotient (MQ) and Trail Making Test Part B (TMT-B). The study indicates that web-based Tai Chi intervention can effectively enhance cognitive function in elderly T2DM patients, outperforming traditional fitness walking and highlighting the potential of remote exercise therapy in cognitive enhancement (107). Sun et al. conducted the first comprehensive evaluation of Tai Chi's effects on metabolic and inflammatory profiles in T2DM patients. The results demonstrated that, compared to usual care or blank control, Tai Chi significantly reduced fasting blood glucose (SMD -0.57), HbA1c (MD -0.73%), triglycerides (SMD -0.50), LDL-C (SMD -0.70), and inflammatory markers including hs-CRP, IL-6, and TNF-α; however, no significant improvements were observed in blood pressure or HDL-C. Subgroup analysis further indicated that the 24-form routine, practiced ≥5 times per week, ≥60 min per session, for ≥12 weeks, yielded optimal glycemic control. The authors propose that Tai Chi exerts its glucose-lowering and anti-inflammatory effects through three complementary pathways: enhanced insulin sensitivity, preserved *β*-cell function, and improved vascular endothelial function, positioning it as a safe, low-cost community-based adjunct exercise therapy for T2DM. The study provides high-quality evidence (rated “high” for HbA1c and “moderate” for other indicators), though limitations include significant heterogeneity in intervention protocols and scarcity of original studies on inflammatory markers. Future research should expand sample sizes, standardize protocols, and extend follow-up periods to validate long-term benefits (108).

From a metabolic perspective, Tai Chi also significantly improves glycolipid metabolism in T2DM patients, which may indirectly support cognitive function. Research has found that 24 weeks of Tai Chi training significantly reduced fasting blood glucose (FBG), low-density lipoprotein (LDL), and glycated hemoglobin (HbA1c) levels in T2DM patients, with superior effects on LDL and FBG compared to the walking group. These metabolic improvements may mitigate the toxic effects of hyperglycemia and dyslipidemia on cerebrovascular function and neurons, thereby slowing cognitive decline (109). Furthermore, Tai Chi can improve insulin sensitivity, potentially helping to alleviate cerebral insulin resistance—a key mechanism in T2DM-related cognitive impairment.

Notably, Tai Chi also significantly ameliorates depressive symptoms in T2DM patients. Studies have shown that after 24 weeks of Tai Chi intervention, patients' Hamilton Depression Scale (HAMD) scores decreased significantly compared to the control group (110). This finding is particularly important as depression is not only common in T2DM patients but can also exacerbate cognitive impairment. By integrating mind-body regulation, Tai Chi may simultaneously address metabolic abnormalities, cognitive decline, and psychological distress in T2DM, achieving multiple benefits. Compared with other forms of exercise, Tai Chi offers unique and comprehensive benefits for patients with T2DM, as it combines low-intensity physical activity with mind-body elements such as breath regulation, balance training, and stress relief. These multidimensional features may simultaneously improve glycemic control, enhance lower limb muscle strength, reduce the risk of falls, and alleviate diabetes-related distress.

### 5.4 Other mind-body medicine traditions

Although Tai Chi is a well-studied example, other forms of mind-body medicine also demonstrate beneficial effects for T2DM complicated with cognitive dysfunction. Ruesi Dadton (RD), also known as Thai Yoga, is a traditional Thai mind-body exercise derived from yoga. It integrates slow movements, deep breathing, self-massage, stretching, and multi-posture balance training, making it well-suited for the physiological characteristics of middle-aged and elderly populations. While no high-quality RCTs have directly investigated its effects in populations with concurrent T2DM and cognitive impairment, three existing RCTs have explored its impact on cognitive function and related biomarkers in patients with MCI and on glycemic and inflammatory markers in individuals with prediabetes. These studies cover core pathological targets of T2DM with cognitive dysfunction—hyperglycemia, chronic inflammation, impaired neuroplasticity, and amyloid deposition—thus providing crucial trans-dimensional evidence for exercise intervention in this condition. The core findings are summarized below in four aspects to meet the inclusion criteria for a review on exercise interventions for T2DM with cognitive dysfunction: (1) cognitive function and neuroprotective biomarkers, (2) regulation of core diabetic pathological indicators, (3) potential mechanisms of action, and (4) commonalities and limitations of the studies (111–113). Furthermore, the beneficial effects of yoga intervention on cognitive function in patients with T2DM and its underlying neurobiological mechanisms are supported by a series of studies. A review by Bali et al. (114) provided a public health perspective, outlining the potential and an application framework for yoga as a comprehensive intervention for managing diabetes and its associated dementia (114). Subsequent randomized controlled trials have furnished specific neurobiological evidence. Specifically, Kaligal et al. (115) found that a 12-week integrated yoga program significantly enhanced prefrontal cortical oxygenation and improved working memory performance in individuals with T2DM (115). The most recent study by Kanthi et al. (116) further corroborated these findings from a neuroelectrophysiological standpoint, demonstrating that yoga practice optimizes event-related potentials in T2DM patients (116). Collectively, this body of literature suggests that regular mind-body exercise, such as yoga, may serve as an effective non-pharmacological intervention for alleviating cognitive dysfunction in T2DM, potentially by improving hemodynamic and neuroelectrophysiological activity in the prefrontal brain regions.

## 6 The mechanism by which exercise therapy improves cognitive dysfunction associated with T2DM

### 6.1 Neurotrophic factors and synaptic plasticity

One of the principal mechanisms by which exercise therapy improves cognitive impairment in T2DM is through the modulation of neurotrophic factor expression and the potentiation of synaptic plasticity. Brain-derived neurotrophic factor (BDNF), the most prevalent neurotrophic factor in the brain, serves a pivotal function in synaptic plasticity regulation, neuronal survival, and differentiation. T2DM patients frequently display reduced BDNF expression in the hippocampus, which is strongly linked to cognitive deterioration.Research indicates that aerobic exercise markedly upregulates hippocampal BDNF levels in T2DM mice, achieving increases of up to two-fold. This elevation in BDNF is strongly associated with heightened expression of the synaptic proteins PSD-95 and SYN, both critical for synaptic integrity and function (117). Behaviorally, the upregulation of BDNF correlates with enhanced performance in contextual fear memory tests, demonstrating that exercise-induced neurotrophic changes translate to meaningful cognitive benefits.Beyond BDNF, exercise also modulates other neurotrophic and growth factors. Studies reveal that physical activity upregulates insulin-like growth factor-1 (IGF-1) in diabetic rats, fostering hippocampal synaptic remodeling (118). Structurally analogous to insulin, IGF-1 not only modulates glucose metabolism but also crosses the blood-brain barrier, where it promotes neurogenesis and synaptogenesis, suggesting a particularly significant role in ameliorating T2DM-related cognitive deficits. Furthermore, studies indicate that aerobic exercise not only improves metabolic parameters but also provides comprehensive clinical benefits for T2DM patients with comorbid mental health issues through multiple mechanisms, such as modulating neuroinflammation, enhancing neuroplasticity, and regulating the expression of neurotrophic factors (e.g., BDNF) (119). Notably, beyond conventional aerobic exercise, other forms of physical activity—particularly mind-body integrated practices—can also effectively elevate levels of neurotrophic factors. For instance, a randomized controlled trial involving patients with MCI demonstrated that a 12-week program of Ruesi Dadton, a traditional Thai mind-body exercise, significantly increased serum levels of BDNF and SIRT1 (111).

### 6.2 Inflammation

Gut microbiota dysbiosis is a significant contributor to neuroinflammation in patients with T2DM. Studies have demonstrated that aerobic exercise can markedly reshape the gut microbial structure in T2DM mice, specifically by increasing the abundance of butyrate-producing bacteria (such as Bacteroidales and Ruminococcaceae), reducing the Firmicutes/Bacteroidetes ratio, and decreasing pro-inflammatory associated bacteria (such as Erysipelotrichaceae and Faecalibaculum). This microbial remodeling enhances intestinal barrier function, restoring the expression of tight junction proteins ZO1 and Occludin from 50% in the diabetic group to 80% of normal levels, thereby effectively reducing endotoxin translocation into the bloodstream and subsequently suppressing neuroinflammation in the hippocampal region. Notably, through fecal microbiota transplantation experiments, researchers transferred the gut microbiota from exercised mice to non-exercised diabetic mice and observed comparable cognitive improvements, directly demonstrating the pivotal mediating role of gut microbiota in exercise intervention (120). Exercise can directly modulate the expression of inflammation-related molecules in the brain. Studies on high-intensity interval training (HIIT) have shown that HIIT intervention significantly upregulates the expression of miR-146a in the hippocampal region of diabetic rats, demonstrating an approximately 42% increase compared to the diabetic group. As a crucial anti-inflammatory microRNA, miR-146a inhibits the release of downstream pro-inflammatory cytokines TNF-α and IL-6 by targeting key adapters IRAK1 and TRAF6 in the TLR4/NF-*κ*B pathway (121). Furthermore, exercise can modulate the activation state of microglia, reducing their polarization toward a pro-inflammatory phenotype, thereby further ameliorating the inflammatory microenvironment in the hippocampal region. These changes are closely associated with improvements in cognitive behavior, such as enhanced performance in contextual fear memory tests.

The anti-inflammatory mechanisms of exercise also involve its regulatory effects on adipose tissue. Eight weeks of high-intensity interval training (HIIT) increased leptin levels in the serum and hippocampus of T2DM rats, while reducing hippocampal levels of BACE1, amyloid-*β*, and hyperphosphorylated tau. Leptin not only attenuates amyloid protein production but also exerts anti-inflammatory effects by modulating the JAK/STAT signaling pathway. Additionally, exercise reduces visceral fat accumulation and decreases the release of pro-inflammatory factors (such as TNF-α and IL-6) derived from adipose tissue, thereby mitigating systemic low-grade inflammation and its impact on blood-brain barrier permeability (122).

### 6.3 Oxidative stress

Oxidative stress represents another core mechanism underlying cognitive dysfunction in T2DM, where chronic hyperglycemia leads to excessive production of reactive oxygen species (ROS), overwhelming endogenous antioxidant defense capacity and resulting in neuronal damage. Exercise therapy addresses this challenge through the activation of multiple antioxidant pathways: Research indicates that exercise effectively activates the Nrf2-superoxide dismutase 2 (SOD2) antioxidant pathway. Nrf2, a key regulator of cellular oxidative stress response, upon exercise-induced activation, promotes the increased expression of downstream antioxidant enzymes such as SOD2, heme oxygenase-1 (HO-1), and glutathione peroxidase (123). These enzymes collectively constitute a robust antioxidant defense system that neutralizes excess ROS and mitigates oxidative damage in hippocampal neurons. In a mouse model of diabetes with concomitant circadian rhythm disruption, exercise intervention not only enhanced antioxidant enzyme activity but also reduced hippocampal lipid peroxidation levels. The optimization of mitochondrial function by exercise also serves as a critical component in counteracting oxidative stress (124). Under T2DM conditions, mitochondrial dysfunction in hippocampal neurons leads to reduced ATP generation and increased ROS production. Regular exercise improves mitochondrial biogenesis and quality control, enhances the efficiency of the electron transport chain, and reduces ROS generation resulting from electron leakage. Exercise can also upregulate the expression of uncoupling proteins (UCPs), thereby alleviating the oxidative stress burden. These adaptive changes collectively protect neurons from oxidative damage and help maintain synaptic plasticity. Studies have found that exercise downregulates mitochondrial ROS production in the hippocampus of diabetic mice while concurrently improving the expression of synaptic proteins PSD95 and SYN. The modulation of oxidative stress-related signaling pathways by exercise involves several key molecules (125). Exercise regulates the SIRT1-GSK3*β* signaling axis; Sirtuin 1 (SIRT1), an NAD+-dependent deacetylase, is activated following exercise intervention (117). Activated SIRT1 further suppresses the activity of glycogen synthase kinase-3β (GSK3*β*), a key mediator that promotes both oxidative stress and neuroinflammation. This regulatory mechanism not only alleviates oxidative damage but also indirectly influences tau protein phosphorylation and insulin signaling pathways, forming a synergistic defense network against oxidative stress.

### 6.4 Autophagy regulation

The PI3 K/Akt/mTOR signaling pathway represents one of the core pathways regulating autophagy. Under T2DM conditions, the PI3 K/Akt/mTOR pathway in the hippocampal region is frequently hyperactivated, thereby suppressing the autophagic process. Studies have demonstrated that both high-intensity interval training (HIIT) and moderate-intensity continuous training (MICT) effectively inhibit the excessive activation of the PI3 K/Akt/mTOR pathway in the hippocampus of T2DM mice, manifested as reduced mTOR phosphorylation levels, which subsequently relieves the inhibition of autophagy and promotes autophagic flux. This regulatory effect induces favorable changes in autophagy markers, including increased Beclin1 expression, elevated LC3-II/LC3-I ratio, and reduced p62 levels. It is particularly noteworthy that the inhibitory effects on mTOR activity vary with exercise intensity. Research indicates that HIIT induces a more pronounced reduction in mTOR activity compared to MICT, potentially attributable to the more substantial metabolic stress elicited by HIIT. From a functional perspective, these changes are significantly correlated with improved spatial learning and memory performance in the Morris water maze test, indicating that exercise-mediated regulation of autophagy via the PI3 K/Akt/mTOR pathway directly contributes to the amelioration of cognitive function in T2DM (126).

In addition to the PI3 K/Akt/mTOR pathway, AMP-activated protein kinase (AMPK), which functions as a cellular energy sensor, also serves as a key molecule mediating exercise-induced regulation of autophagy. Exercise depletes cellular energy reserves, leading to an increased AMP/ATP ratio and subsequent activation of AMPK. The activated AMPK directly phosphorylates the forkhead box O transcription factor FoxO3a, promoting its nuclear translocation and thereby regulating the expression of downstream autophagy-related genes such as Bnip3 and Spk2. Bnip3, a critical regulator of mitophagy, enhances LC3 expression and upregulates autophagic activity. Concurrently, the AMPK-FoxO3a axis indirectly promotes autophagy by suppressing mTORC1 activity. This pathway plays a significant role in exercise-induced amelioration of cognitive dysfunction in T2DM, with FoxO3a being recognized as a potential therapeutic target. Studies indicate that exercise-induced AMPK activation not only directly promotes autophagy but also improves mitochondrial function and reduces oxidative stress, thereby protecting hippocampal neurons from hyperglycemia-induced damage (123, 127).

Aerobic exercise, one of the most extensively studied forms of physical activity, has been well-documented for its role in modulating autophagy. It has been demonstrated to significantly improve cognitive function in diabetic rats, as evidenced by shortened escape latency and increased platform crossings in the Morris water maze test. At the molecular level, aerobic exercise upregulates the expression of brain-derived neurotrophic factor (BDNF) and cAMP response element-binding protein (CREB) in the hippocampal region, while modulating cell cycle and apoptosis-related proteins (e.g., reducing CDK5, cyclin D1, and Caspase-3), thereby improving the neuronal microenvironment. The regulation of autophagy by aerobic exercise is characterized by an overall enhancement of autophagic flux, encompassing the entire process from autophagy initiation and autophagosome formation to substrate degradation. Studies have shown that aerobic exercise significantly increases the LC3-II/LC3-I ratio and reduces p62 protein levels in the hippocampus of diabetic mice, indicating enhanced autophagic activity (128). Furthermore, aerobic exercise can alleviate inflammatory responses and apoptosis in hippocampal tissue by modulating miR-126 expression, indirectly influencing the autophagic process.

In contrast to aerobic exercise, the effects of resistance exercise (e.g., ladder climbing training) on autophagy regulation have been less extensively investigated. However, existing evidence suggests that it is similarly effective in ameliorating cognitive dysfunction in T2DM (129, 130). Research has found that resistance exercise significantly reduces microglial activation (indicated by decreased Iba-1-positive cells) and NLRP3 inflammasome activity in the hippocampus of diabetic mice, while increasing the expression of Arg-1, a marker of M2-type microglia. These changes are closely associated with autophagy activation. Notably, combined exercise (integrating aerobic and resistance training) may yield more pronounced effects in regulating autophagy and improving cognitive function. A comparative study of treadmill exercise (aerobic), ladder climbing (resistance), and their combination (combined) revealed that combined exercise produced the most significant modulation of autophagy-related indicators (e.g., reduced Bax and p62 protein expression, increased Bcl-2 and LC3 protein expression) (99, 131). From a functional perspective, the combined exercise group exhibited the best performance in the Morris water maze test, suggesting that different exercise modalities may synergistically and more effectively activate autophagy, thereby improving cognitive function.

### 6.5 Gut-brain axis regulation

Cognitive dysfunction associated with T2DM has been confirmed to be closely related to functional disruption of the gut-brain axis. The gut-brain axis represents a complex bidirectional communication network between the gut and the brain, involving multiple pathways such as neural signal transmission, immune-inflammatory regulation, and metabolic exchange. Under T2DM conditions, chronic hyperglycemia and insulin resistance can trigger gut microbiota dysbiosis, compromise intestinal barrier integrity, and facilitate the entry of bacterial toxins and inflammatory factors into the systemic circulation. This subsequently disrupts the blood-brain barrier, inducing neuroinflammation and neuronal damage. Studies have shown that both T2DM patients and animal models exhibit significant alterations in gut microbiota composition, characterized by an increased Firmicutes/Bacteroidetes ratio, reduced abundance of butyrate-producing bacteria, and an increase in conditional pathogens. This microbial imbalance impacts cognitive function through multiple mechanisms: on one hand, decreased levels of short-chain fatty acids (SCFAs), particularly butyrate, impair their anti-inflammatory and histone deacetylase inhibitory functions; on the other hand, increased pathogen-associated molecular patterns can activate systemic and central immune responses, leading to impaired synaptic plasticity and reduced neuronal survival in the hippocampal region. Furthermore, alterations in gut microbiota also affect the function of the microbiota-metabolite-brain axis, further exacerbating cognitive impairment (120). Research in mouse models of diabetic cognitive dysfunction has revealed significantly reduced levels of several beneficial microbial metabolites, such as 3-indolepropionic acid, 5-hydroxytryptamine, and SCFAs, which are crucial for maintaining mitochondrial function, neurotransmitter balance, and energy metabolism. Consequently, interventions targeting the gut-brain axis, particularly exercise therapy, have emerged as novel strategies for preventing and treating T2DM-related cognitive decline (119, 132).

A randomized controlled trial involving postmenopausal women with T2DM demonstrated that 12 weeks of home-based multi-task exercise training significantly increased the abundance of Akkermansia muciniphila and Faecalibacterium in the gut, while reducing Lactobacillus levels. Notably, the increased abundance of Akkermansia muciniphila was positively correlated with improved high-density lipoprotein levels and enhanced cognitive function, suggesting that this bacterium may play a pivotal role in exercise-induced neuroprotection (133). The regulation of gut microbiota by exercise exhibits a clear dose-response relationship, with moderate exercise providing optimal benefits. One study revealed that moderate exercise promotes cognitive function and hippocampal neurogenesis through gut microbiota modulation, whereas high-intensity or prolonged exercise can reverse these benefits. This biphasic effect exemplifies exercise-induced hormesis, a phenomenon where low doses are beneficial, but high doses are detrimental (134). The regulatory effects of exercise on gut microbiota also vary with exercise modality. High-intensity interval training has been confirmed to significantly improve gut microbiota composition in T2DM patients, increasing the relative abundance of Bacteroidetes, Actinobacteria, Proteobacteria, and Fusobacteria, while reducing the relative abundance of Firmicutes—particularly diminishing Ruminococcus torques and Ruminococcus gnavus, which are positively associated with glucose metabolism abnormalities (135). These microbial changes occurred concurrently with improvements in glucose metabolism indicators, suggesting that high-intensity interval training may exert multiple benefits through the microbiota-metabolism axis.

## 7 Challenges and future directions

Although exercise therapy shows considerable promise in the management of T2DM with cognitive impairment, several challenges and unresolved issues remain. First, current evidence is limited by inadequate sample representativeness, lack of intervention standardization, and insufficient long-term follow-up. For example, most studies have been conducted in specific regional populations, which may limit generalizability, and variability in exercise protocols—such as 30-minute Tai Chi vs. 60-minute walking—complicates cross-study comparisons. Second, the influence of individual differences on intervention outcomes warrants further investigation. Future studies should aim to identify reliable biomarkers capable of predicting exercise responsiveness, enabling the design of tailored and optimized exercise regimens for individual patients. Moreover, the synergistic potential and optimal combination of different exercise modalities remain to be clarified. Furthermore, in writing the present review, only reliable data sources such as Scopus, Web of Science, PubMed, and ScienceDirect were selected, while relevant studies indexed in other databases were not adequately retrieved. For the retrieved literature, we merely classified them based on their content (aerobic exercise, resistance exercise, Tai Chi), without systematically reviewing the development of various exercise therapies from a temporal perspective.

Future research should prioritize the following areas: (1) conducting large-scale, long-term follow-up studies to evaluate the effect of exercise interventions on dementia conversion; (2) elucidating underlying mechanisms using multimodal neuroimaging and molecular biology techniques; (3) developing personalized exercise prescriptions based on genetic profiles, clinical phenotypes, and lifestyle factors; (4) exploring optimal integration of exercise with other interventions, such as pharmacotherapy and cognitive training; and (5) incorporating smart health technologies to establish remote monitoring and guidance systems, thereby improving feasibility and adherence.

From a translational perspective, future efforts should address how to effectively incorporate evidence-based exercise interventions into routine clinical and community healthcare systems. As a low-cost, readily scalable non-pharmacological intervention, Tai Chi could be integrated into clinical guidelines for diabetes and MCI management, particularly in resource-limited primary care settings. Combined with tele-rehabilitation technologies, it holds potential for expanding population coverage and facilitating the individualized promotion of “exercise prescriptions.”

## 8 Conclusion

Exercise therapy represents a non-pharmacological intervention for T2DM-associated cognitive dysfunction, offering unique advantages including multifaceted benefits, low cost, and a high safety profile. Current evidence indicates that various exercise modalities—such as aerobic exercise, Tai Chi, and dual-task training—can improve cognitive function in T2DM patients to varying degrees. Among these, Tai Chi, characterized by its integrated mind-body practice, demonstrates comprehensive advantages in enhancing cognition, balance, and sleep quality.These exercise regimens exert their effects through multi-level, multi-system mechanisms. These include molecular-level upregulation of BDNF and reduction of inflammatory factors, cellular-level enhancement of synaptic plasticity, system-level improvements in gut-brain axis regulation and insulin sensitivity, and brain network-level optimization of functional connectivity. These mechanisms collectively form the scientific foundation for the beneficial role of exercise in mitigating cognitive decline in T2DM.Future research should focus on developing personalized exercise prescriptions, optimizing the combination of different exercise modalities, and exploring synergistic effects with other interventions. By integrating modern technologies such as mobile health and remote monitoring, it will be possible to advance the precision and accessibility of exercise interventions, thereby maximizing the delay of cognitive decline and improving the quality of life for individuals with T2DM.
